# Mortality and demographic recovery in early post-black death epidemics: Role of recent emigrants in medieval Dijon

**DOI:** 10.1371/journal.pone.0226420

**Published:** 2020-01-22

**Authors:** Pierre Galanaud, Anne Galanaud, Patrick Giraudoux, Henri Labesse

**Affiliations:** 1 Université Paris-Saclay, Inserm, Inflammation, Microbiome and Immunosurveillance, Clamart, France; 2 Université de Bourgogne-Franche-Comté, Besançon, France; 3 Chrono-environnement, Université de Bourgogne-Franche-Comté, CNRS UMR6249, Besançon, France; 4 Paris Sorbonne—Paris 4 Université, Institut des Sciences Humaines Appliquées, Paris, France; University of Louvain, BELGIUM

## Abstract

**Objective and methods:**

We analyze the influence of population movement on susceptibility to death and resilience during two epidemics occurring in Dijon soon after the Black Death. Using a specific program designed to propose links between entries in annual tax registers, we define tentative heads of household, the elapsed time since their first registration and their ties with other persons within the city.

**Results:**

During the 1400 epidemic heads of household who were registered for 1–3 years die in large numbers, whereas during years without epidemics, their death rate is lower than that of heads of household who were registered longer. Recent registration is an epidemic vulnerability factor only in association with a low taxation status, which, when isolated, does not influence mortality. A lack of familial ties within Dijon is another vulnerability factor among the recently registered. This suggests that poor, recent emigrants are more affected by epidemic mortality. In contrast, the mortality of recently registered heads of household is indistinct during a later epidemic occurring after several years of major famine that may have selected the more resistant emigrants and/or excluded the more miserable of them from our analysis. In contrast to the first one, this second epidemic is followed by rapid demographic recovery. This latter recovery is fully explained by the contribution of poor, newly registered heads of household without ties in Dijon.

**Conclusion:**

Our results outline the interaction between population movement and low socioeconomic status on death susceptibility in historical plagues and show that poor recent emigrants may also be key players in the resilience of the population after an epidemic.

## Introduction

Plague, recognized as a present-day re-emerging health threat [1], is one of the most extensively studied communicable diseases of the past. In Europe, the deadly mid-14th century Black Death and its multiple recurrences until the middle of the 18th century [2–7] have been recorded in numerous documents and *Yersinia pestis* has been characterized in human remains from victims of several of these epidemics [8–12].

The characterization of a draft genome of *Yersinia pestis* from victims of the Black Death demonstrated that the role of host susceptibility has to be considered in the epidemiology of historical infections [9]. Taking into account host susceptibility has brought back a long-standing debate about death selectivity during historical plagues [4, 13, 14]. The influences of frailty, age, gender, and socioeconomic status on plague-related mortality were recently reevaluated by paleodemographic methods [15–18] and by the direct and extensive analysis of historical sources [19, 20] combined with the calculation of fatality rates [21] or of individual's risk of death [22]. This has contributed to a better knowledge of the disease despite a number of conflicting results. These discrepancies are possibly attributable to the heterogeneity of the epidemics under study and/or to the non-exhaustive nature of the documentation with respect to a number of critical and possibly interactive frailty factors.

Population movement is one factor that may influence resistance to epidemic mortality. Indeed, emigrants were present among the urban victims of plague epidemics [19, 21, 23]. The vulnerability of newcomers may be conditioned by factors such as their usually lower socioeconomic status and/or housing conditions. Their vulnerability may also reflect the consequences of population movement itself such as suboptimal social bonds. Determining the role of population movement in historical epidemics is important because emigration towards urban centers was already significant in the medieval era [24]. This may have implications for present-day plague outbreaks, which tend to occur in conjunction with other disasters [25] that often represent an incentive to move. To determine the influence of having recently settled somewhere else on plague-related death, we undertook an analysis of factors influencing mortality during epidemics occurring soon after the Black Death in the city of Dijon.

Our study is mainly based on the annual registers of the *marcs* tax accounts (*marcs* registers), which provide information on the demography of heads of household in late medieval Dijon with an exceptional continuity over time from the mid-fourteenth century to the early sixteenth century [26, 27]. Registers were continuously available for several years before and after the two epidemics selected for this study, corresponding to the mortality crises in 1400 and 1438–1439. To exploit this opportunity, we used a program designed to identify tentative individual heads of household as persons (with defined dates of first and last registration) from annual entries of households in the tax registers [28]. This allowed us to select heads of household according to the time elapsed between their first registration and the epidemic. The program also highlights the relationships between persons included in the database. We can thus determine whether an individual head of household had a familial link with another person. Recently registered heads of household without a demonstrable tie within Dijon were selected by combining the delay from first registration and interpersonal relationships. Our hypothesis was that this group includes a higher proportion of newcomers, thus providing an approach to the differential vulnerability of heads of household recently settled in the city. Using the same criteria for the individualization of newcomers, we extended the analysis to determine their contribution to demographic recovery during post-epidemic years, which is a poorly studied aspect of historical epidemics.

## Materials and methods

### Historical sources

The main historical sources are the *marcs* registers from Dijon that annually recorded heads of household in order to levy the *marcs* tax. The registers indicate the names of the heads of household, the amount of tax they paid or their exemption from taxation, their death or departure, their home location (parish and street) and in some cases their profession [26, 27, 28]. For this work, 50 annual *marcs* registers, reflecting the demography of the years from 1375 to 1446, were taken into account **(S1 Text)**.

### Database

The database was created from this unique historical source and based on an original program specially designed for a previous study [28]. The program allowed us to propose links between the more than 100,000 annual entries of households so as to define tentative individual heads of household identified as persons with an ID number, a standardized name, a year of first registration in the registers and a year and mode of disappearance from them **(S2 Text)**.

Among the 13,001 heads of household identified, 12,874 corresponded to physical persons who were suitable for mortality analysis and were selected for this work **(S3 Text)**.

In addition, 1,980 persons related to heads of household (when mentioned in the registers or found in other source materials) were also included in the database and given an ID number **(S4 Text)**. Although not eligible for demographic analysis, these persons were taken into account when determining the relationships the heads of household had with other people within Dijon (see below).

### Demographic information from the registers

During the period under study approximately 2,000 individual heads of household were registered annually in the *marcs* registers. The proportion of registered female heads of household regularly decreased, from 17% in 1400 to 7.5% in 1438 and 9.4% in 1439. The enlistment in the *marcs* registers was not limited to the heads of household who were subject to the *marcs* tax, as those exempted from this tax were also included in the registers. However, the number of heads of household indicated in the *marcs* registers is an underestimate of the inhabitants for the following reasons. (i) A head of household was registered for his/her whole family (including servants in some cases). (ii) Although heads of household exempted from the *marcs* tax were recorded in the registers, the registration of these citizens not subject to the tax was possibly less exhaustive. (iii) The registers enrolled essentially solvent heads of household, neglecting most of the very poor, as well as vagabonds and beggars **(S5 Text)**.

In the registers, the heads of household who were still present were taxed or exempted. Those who were no longer present were marked with a mention of their death or of absence, and the amount of their tax was crossed out. A number of absent individuals were in all probability still alive in the year of their reported absence **(S6 Text)**. From year to year, a number of heads of household were no more mentioned in the registers. In some instances, their death could be documented by the mention of their widow (either enlisted as a head of household at the same place or mentioned as the new wife of another head). In this case they were included as dead in the corresponding year of the database. Otherwise they were considered as lost to follow-up. The proportion of these lost to follow-up was marginal (0.9% in 1400, 1.9% in 1438 and 2% in 1439). The crude death and absence rates were estimated by the percentage of deaths or absences compared to the total number of household entries in the register [dead + absent + alive (whether present or absent but still alive)]. The time elapsed since the first registration of each head of household (time since registration) was determined from the database.

For the one-year follow-up, the heads of household reported as present in the year preceding the epidemic were individualized. Their fate in the register of the following year was determined (dead, alive, absent or lost to follow-up) **(S7 Text)**.

### Years of epidemic

Years of epidemics were selected on the following basis: (i) a death rate ≥ 5% in the *marcs* register (the upper 95% confidence limit of the death rate in the 6 registers preceding the 1400 epidemic register was 4.8%); (ii) the availability of several years of registers before and after the mortality crisis; and (iii) a plague report in historical sources [5, 6]. The 1400 epidemic reflects a major post-Black Death plague recurrence that was generalized in Europe [6]. In Dijon, the death rate of the 2,149 registered heads of household was 15.1%, which was comparable to that of one of the last great European plagues [22]. The second epidemic developed over two years [6, 29, 30], in 1438 (with a death rate of 5.5% among the 1,951 registered heads of household) and in 1439 (with a death rate of 10.4% among the 1,814 registered heads of household), resulting in a cumulated death toll comparable to that of 1400. This epidemic occurred after several years of devastating and generalized starvation [31] and climate variability [32].

### Time distribution of the registers and first registration

**Fig 1:**
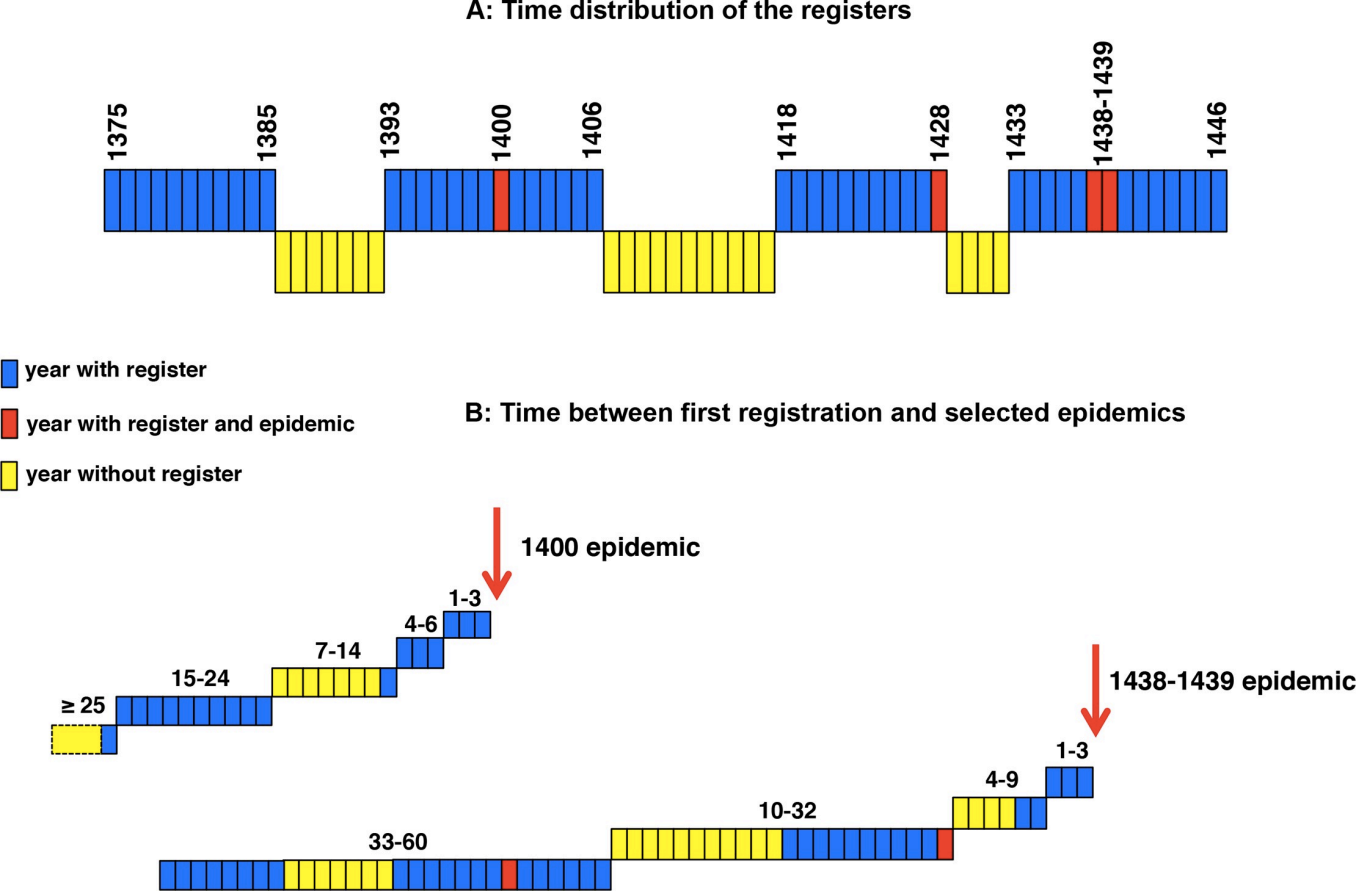
Time distribution of the registers and first registration. Blue boxes: years with registers; red boxes: years with registers and epidemic; yellow boxes: years without registers. Panel A: Time distribution of the registers. Panel B: Time between first registration and selected epidemics. The heads of household first present in 1375 had been registered in 1375 or earlier. Those first present in 1393, 1418 or 1433 had been registered between 1386 and 1393, 1407 and 1418 and 1429 and 1433 respectively. For the 46 other years, the year of first registration corresponds to the register of their first appearance.

The 50 registers included in the database are grouped according to four continuous series spread over 72 years (1375 to 1385, 1393 to 1406, 1418 to 1428 and 1433 to 1446). A diagram of this time distribution outlining the years of epidemics is presented in Fig 1A. Due to the discontinuities in the succession of available registers it was impossible to precisely determine when the first registration took place for heads of household appearing in the first year of the database or in the first year of one of the other three continuous series (Fig 1B). The guidelines used for the analysis of time since registration are presented in **S8 Text**.

### Socioeconomic information from the *marcs* tax

The *marcs* tax level of each head of household was used as an indicator of his/her wealth status. For each taxpayer, the amount of the *marcs* tax stood between an upper limit of 120 sols and a lower limit of 1 sol. Approximately 10% of the taxpayers were charged at or above 20 sols; these taxpayers charged at the top decile were considered high taxpayers in this study. Approximately half of the taxpayers were charged at 1 sol; these taxpayers taxed below the median were considered low taxpayers in this study **(S9 Text)**.

Fewer than 10% of the registered heads of household were exempted from the *marcs* tax, most of them by privilege and a minority because of poverty. The small number of registered heads of household who were exempted on the basis of poverty were grouped with the low taxpayers for the analysis **(S10 Text)**.

### Relationships between persons

The program takes into account the relationships between different persons included in the database. Links between persons are systematized and, when relevant, enriched (such as highlighting an indirect link through a third party). Once identified, relationships were identified by the ID numbers of the persons concerned and by an identification code for the type of link. By querying the database one can find the relationships that each head of household may have had with other persons.

This procedure was used to search for familial ties between heads of household and other persons within Dijon. Heads of household having a detectable relationship with another person in the database were selected, and the nature of the relationship was determined. When necessary, a search was made in the database for more precise information, such as the date in the case of marriage. The following relationships were taken into account for the assessment of having ties within Dijon: (i) Having familial relationships such as "son of", "widow of", "spouse of", or "son-in-law"; (ii) Getting married to a person previously registered or known to be present in Dijon; and (iii) Being the heir of a person in Dijon **(S11 Text)**.

For mortality analysis, relationships were considered for heads of household with a time since registration of 1 to 3 years or of at least 15 years at the time of the 1400 epidemic. For the analysis of the putative origin of new heads of household in pre- and post-epidemic years, relationships were considered for those newly registered (and alive) in each of the years under study.

### Data extraction and statistical analysis

The database allows a longitudinal analysis of individual heads of household or of cohorts of heads of household, as well as a transversal analysis focused on a year of interest **(S12 Text)**.

The informative data corresponding to each year of interest were selected from the database and exported as Excel files for analysis and statistical tests as exemplified in **S1 Table** for folios 1r and 1v of year 1400. A picture of folio 1r of the corresponding source document is presented in **S1 Fig**. A picture of folio 1v was already published [29].

A chi-square test, or a Fischer test for small samples, was used to test the null hypothesis of categorical variable independence in contingency tables. The Wilcoxon signed-rank test was used to compare matched data. For multivariate analysis, mortality was modeled with status (dead = 1, non-dead = 0) as a response variable against independent variables using the general linear model (GLM) with a binomial (logit) link function. Computing and graphical display were performed using Excel 2011 and R 3.6.0 (R Core team 2019).

## Results

### The 1400 epidemic: Time since registration and mortality at one-year follow-up

**Fig 2:**
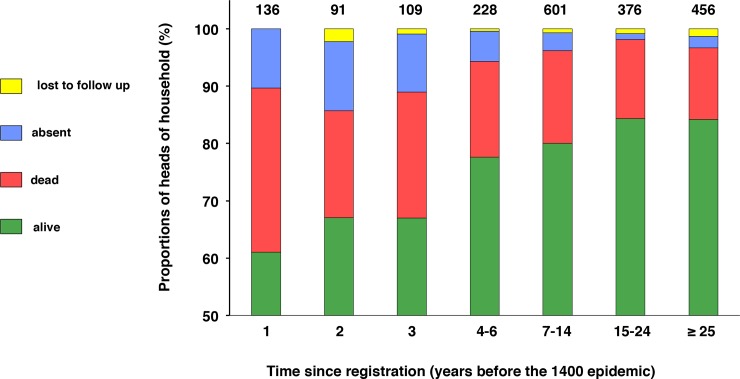
Time since registration and mortality at one-year follow-up (1400 epidemic).

To approach the impact of time since registration, we carried out a one-year follow-up of the heads of household present in 1399, the year preceding the 1400 plague. In the "year of the plague", their situation was as follows: 16.2% were dead, 78.9% were alive, 4% were absent and 0.9% was lost to follow-up. Their fate varied by time since registration (**Fig 2**). The proportion of those who were deceased was higher for those with a shorter time since registration. This was also the case for the proportion of those who were absent. The proportion of those lost to follow-up was low (0 to 2%) and without relation to time since registration.

Time since registration: distributed as shown in Fig 1B, except that times of 1–3 years were split into 1, 2 and 3. Time since registration was estimated with respect to the "year of the plague" (a head of household with a 1-year follow-up was registered in the year preceding the epidemic). For each group of time since registration, the proportions of heads of household alive, dead, absent or lost to follow-up were expressed as percentages of the total number of those present and alive in the year preceding the epidemic. The total numbers are shown on the top of each histogram and the data are shown in actual numbers in **S2 Table**. Collapsed categories 1–3 differed from collapsed categories 4–6 and older for those who were dead, absent or alive (chi-square test; p < 0.0001).

Thus, in the "year of the plague" heads of household whose first registration took place during the three years preceding the epidemic exhibited a higher mortality rate as well as a higher rate of absence. The heads of household with a time since registration of 1, 2 or 3 years were qualified as recently registered in this study and, unless otherwise stated, were collectively analyzed. The other heads of household were qualified as having been registered longer.

### The 1400 epidemic: Time since registration and death rate

**Fig 3:**
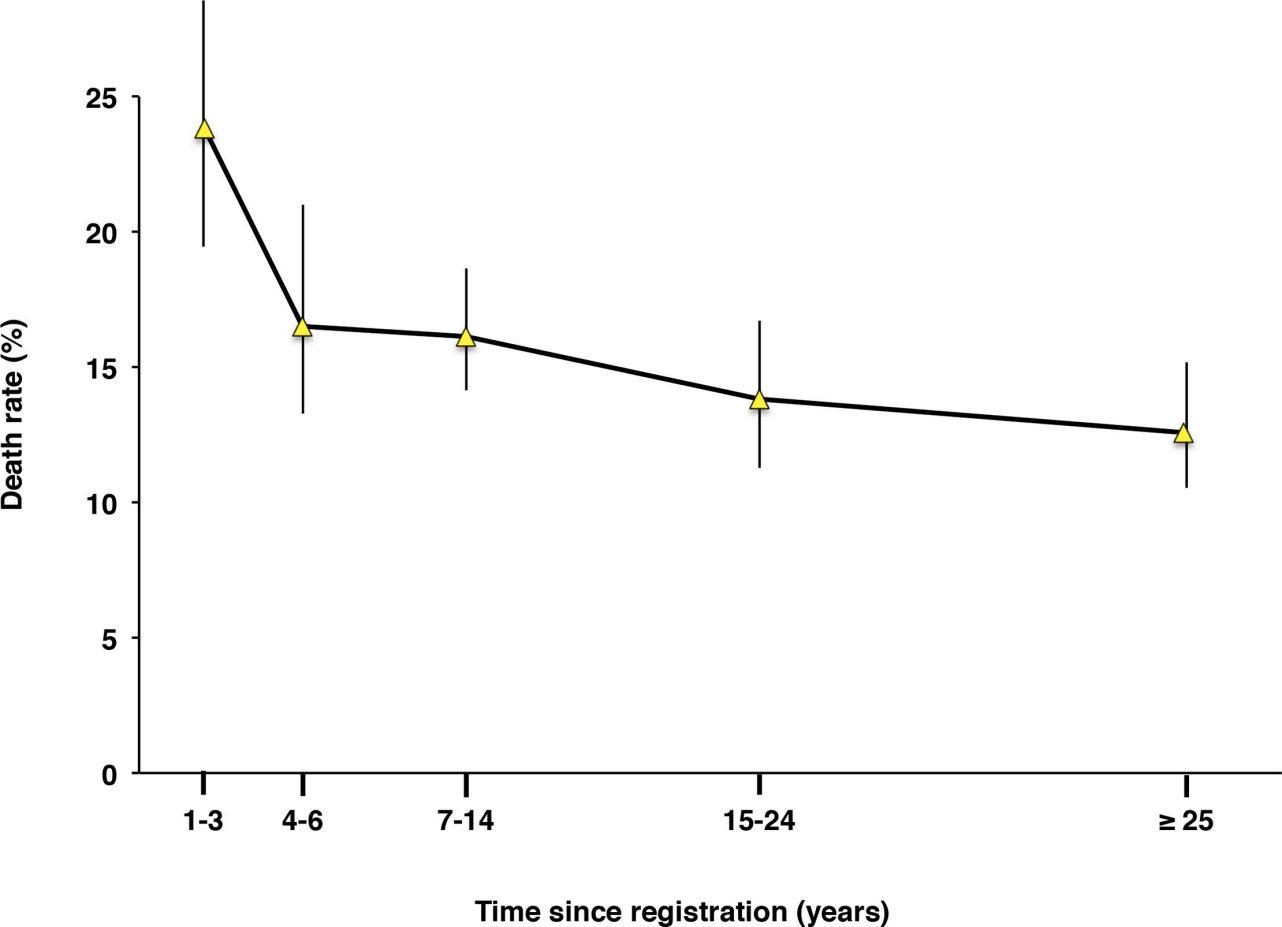
Time since registration and death rate (1400 epidemic). Time since registration: distributed as shown in Fig 1B. Death rate: number of deaths of heads of household/total number of household entries in the register. The vertical bars are 95% confidence intervals. Categories 1–3 showed significantly higher death rates than the collapsed categories 4–6 and older (chi-square test; p < 0.0001). The data are shown in actual numbers in **S3 Table**.

The heads of household listed in the register corresponding to year 1400 were grouped according to their time since registration and their death rates were compared (Fig 3). The recently registered heads of household died at a higher rate than those heads who had been registered longer. For heads of household whose first registration took place more than three years prior to the epidemic year, time since registration did not influence the death rate, even for those who were present in the registers at least 25 years prior.

## Before, during and after the 1400 epidemic: Time since registration, mortality and absence

The higher mortality of recently registered heads of household might reflect a permanent fragility of this group or reveal the group's selective vulnerability during the epidemic. We thus compared the death and absence rates of recently registered heads of household with those of heads who had been registered longer during three different periods of time: during the "year of the plague", during the three years preceding the plague and during the three years following the plague. For the years preceding and following the plague, the results of the three years were grouped in view of the small number of deaths during "regular" years.

**Fig 4:**
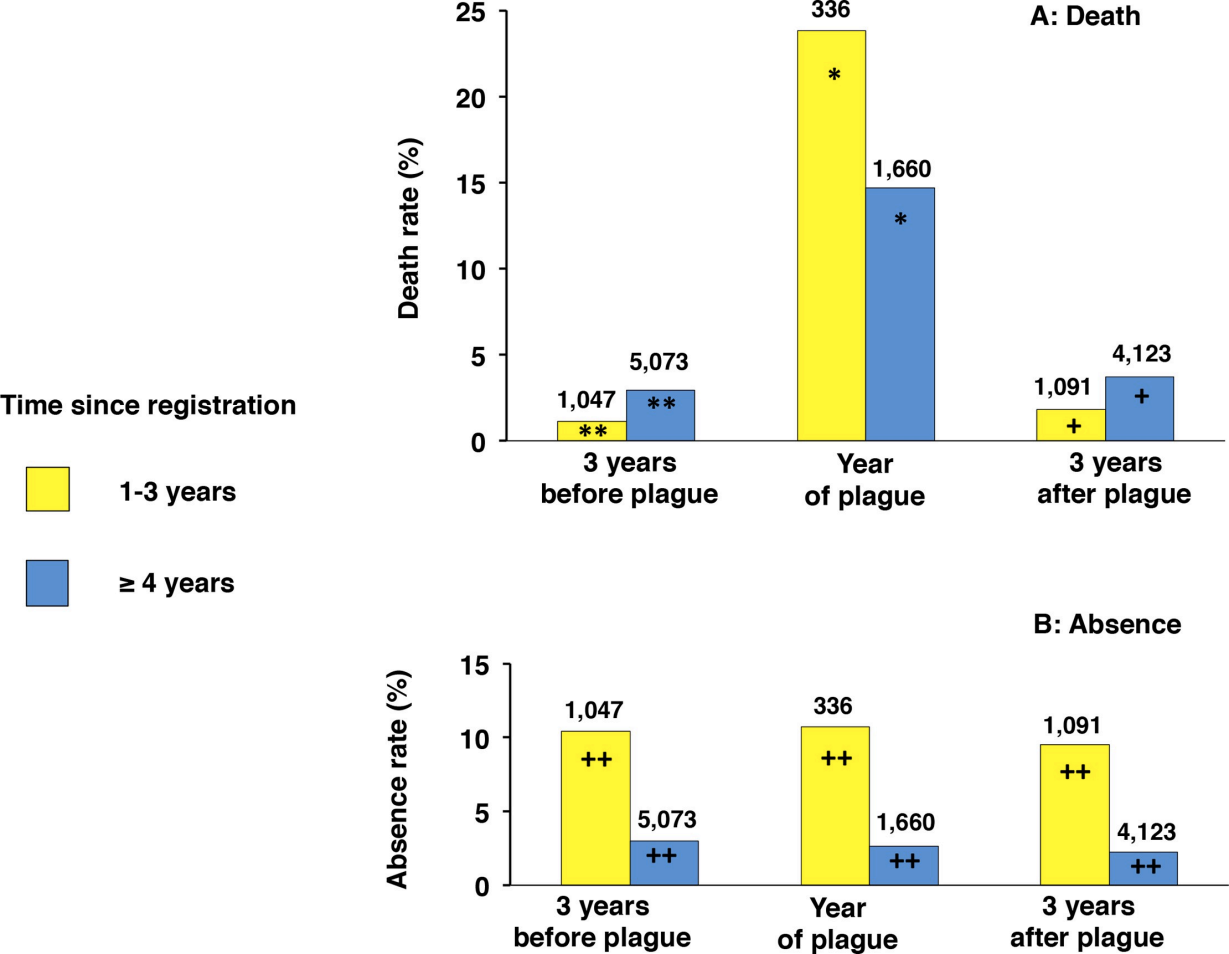
Time since registration, death and absence before, during and after the 1400 epidemic. Panel A: results for mortality; panel B: results for absence. The death or absence rate is given as the percentage of the total number of household entries in the registers during three periods: the three years preceding the plague (cumulated total number); the "year of the plague"; and the three years following the plague (cumulated total number). For each period, two groups of time since registration were taken into account: 1–3 years and ≥ 4 years. Time since registration was determined from the date of first registration with respect to the year when absence and mortality were taken into account. The total numbers of household entries are indicated on top of each histogram. * chi-square test; p < 0.0001. ** chi-square test; p = 0.0009. **+** chi-square test; p = 0.0018. **++** (for all categories) chi-square test; p < 0.0001.

When mortality was considered (Fig 4A), during the "year of the plague" the heads of household with a time since registration of 1–3 years died in greater numbers than did those who had been registered longer (23.8% compared to 14.7%). In contrast, during the years preceding and following the plague, they died in fewer numbers than the more long-term registrants, 1.1% and 3%, respectively, before the epidemic and 1.8% and 3.7%, respectively, after the epidemic.

During these "regular" years, the lower death rate of the recently registered was paralleled by a higher death rate of those registered the longest (**S13 Text** and **S2 Fig**).

When absence was considered (Fig 4B), the heads of household with a time since registration of 1–3 years tended to be more frequently absent. However, in contrast with the percentage of deaths, the percentage of those absent was not increased during the "year of the plague".

Thus, the higher mortality of recently registered heads of household was selective for the year 1400, whereas their rate of absence was not affected by the epidemic.

### The 1400 epidemic: Tax level, time since registration and mortality

As wealth status may influence epidemic mortality, we tested whether tax level played a role during the 1400 epidemic and examined its distribution among recently and more long-term registered heads of household. The death rate of high taxpayers was without particularity (16.1%, compared to 16.2% for their fellow citizens; N = 180 and N = 1,816, respectively). Low taxpayers died at a higher rate (19.3%, compared to 13.4% for non-low taxpayers; N = 965 and N = 1,031, respectively; chi-square test; p = 0.0004). In contrast, during "regular" years, the death rate of low taxpayers was without singularity (2%, compared to 3% during the three years preceding the epidemic; N = 3,232 and N = 3,295, respectively). When recently registered and more long-term registered heads of household were compared, the former included more low taxpayers than the latter (74.7%, compared to 43%; N = 336 and N = 1,660, respectively; chi-square test; p < 0.0001).

**Fig 5:**
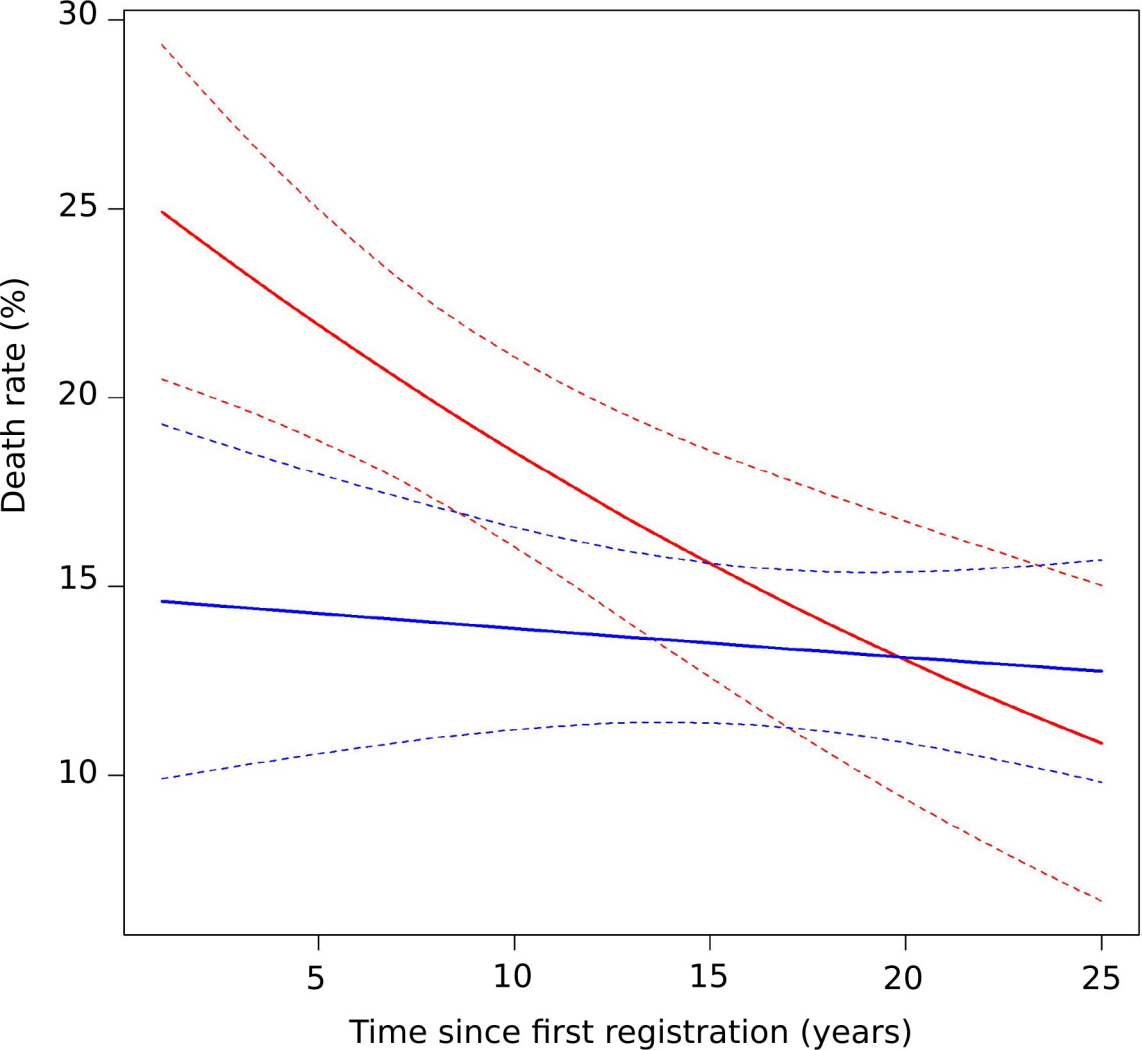
Mortality in relation to time since registration and fiscal category during the 1400 epidemic. Red line: low taxpayers. Blue line: non-low taxpayers. Dotted lines: 95% confidence interval limits.

We thus compared the impact of time since registration on the death rate of low taxpayers and of non-low taxpayers. The death rate was modeled against wealth status and time since registration using a binomial GLM (Fig 5 and **S4 Table**). For low taxpayers, the death rate was significantly higher for the more recently registered, whereas for non-low taxpayers, time since registration had no impact on mortality. The difference in mortality between taxpayer categories decreased with time since registration: mortality was 1.7 times higher for low taxpayers who had been registered for 1 year and was similar between the two categories after 10 years. The statistically significant interaction between time since registration and taxpayer categories **(S4 Table)** materialized by a difference in slope between the two curves (Fig 5), indicates that the death rate varies differently among low taxpayers and non-low taxpayers.

We thus identified a population of heads of household who were highly susceptible to epidemic mortality, characterized by a *marcs* tax level at the tax floor and by recent registration in the annual *marcs* registers. Their vulnerability was not related to their home location **(S14 Text)**. Their professional activities, although slightly different from those of more long-term registered heads of household, were not particularly at risk during epidemics **(S15 Text)**.

### The 1400 epidemic: Vulnerability of newcomers

**Fig 6:**
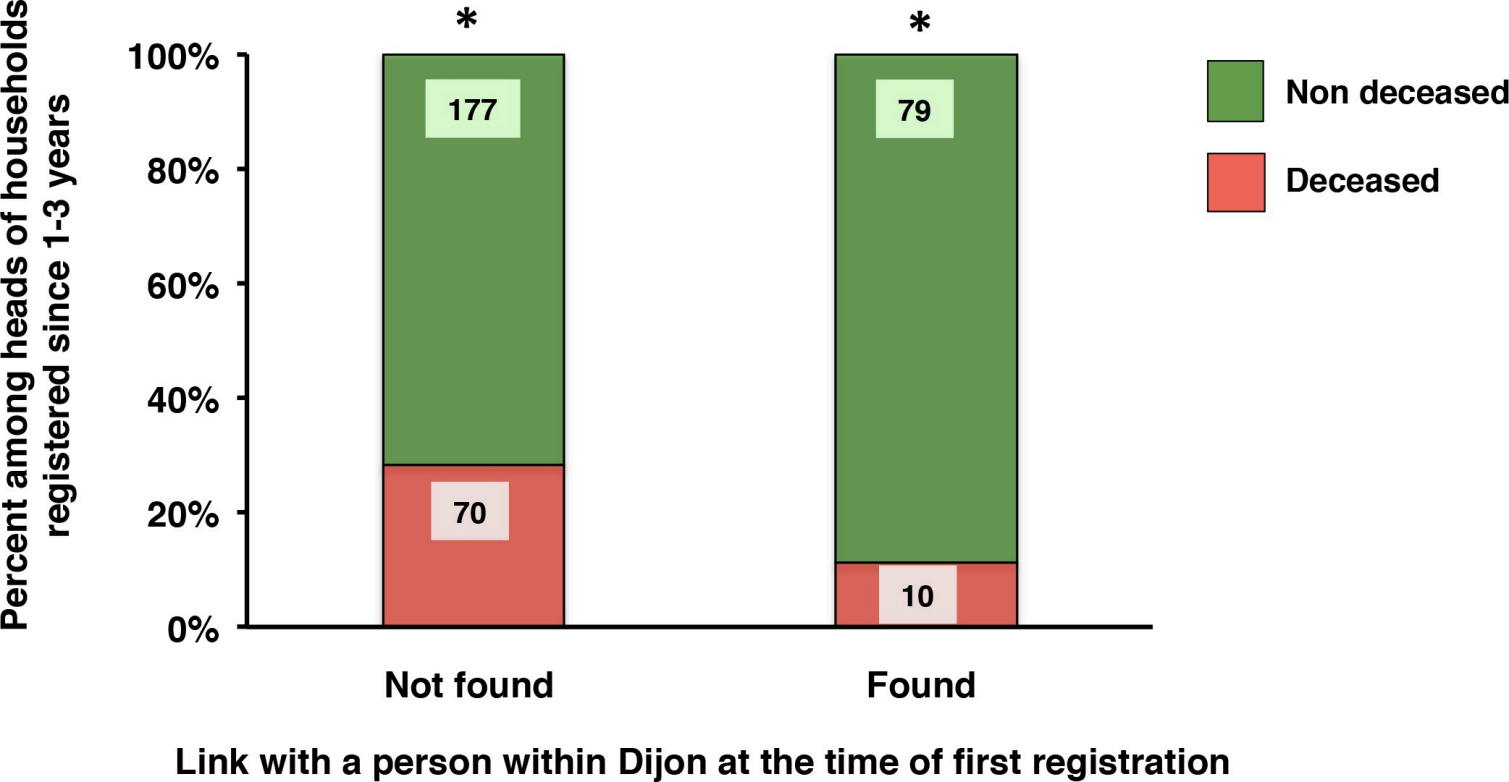
Mortality of recently registered heads of household during the 1400 epidemic according to their links with other persons within Dijon. Mortality: percentages of deceased and non-deceased heads of household with a time since registration of 1–3 years, according to the absence (not found) or presence (found) of a demonstrable link with a person within Dijon. The actual numbers are indicated in each part of the histograms. * chi-square test; p = 0.0012.

We thus tested whether characteristics potentially associated with the status of a newcomer might enhance the vulnerability of recently registered heads of household. Newcomers are less prone to having ties, such as familial links within Dijon at the time of registration. The recently registered heads of household for whom no link was found with other persons from Dijon displayed a higher mortality than did the group with documented links (Fig 6).

Heads of household without demonstrable links included more low taxpayers (86%, compared to 45% among those with links; N = 247 and N = 89, respectively). In view of the differential vulnerability of low and non-low taxpayers, we performed an additional analysis restricted to low taxpayers among recently registered heads of household. Again, those without demonstrable links displayed a higher death rate (30.2%, compared to 12.5%; N = 212 and N = 40, respectively; chi-square test; p = 0.021). This strongly suggests that the effect of links is not the result of a bias attributable to the higher proportion of low taxpayers among them.

In addition, the higher mortality of heads of household without a demonstrable link was particular to recently registered heads of household. Indeed, among the more long-term registered heads of household (first registered between 1375 and 1385), the death rate of those without a demonstrable link was not significantly higher (13.4%, compared to 10.4%; N = 754 and N = 77, respectively; chi-square test; p = 0.46).

Having a place name as a last name is also suggestive of a recent emigration into the city. In 1400 approximately half of the heads of household (48%) had a place name as their last name, which suggested an origin in Burgundy for more than 80% of the heads. As a whole, the death toll was comparable for those with and those without a place name (16.4% and 13.9%, respectively; N = 980 and N = 1,016, respectively; chi-square test; p = 0.28), with the striking exception of those registered in 1399, among whom 39.6% of those with a place name died, compared to 22.7% for their fellow citizens (N = 48 and N = 88, respectively; chi-square test; p = 0.038).

The one-year follow-up of heads of household present in the year preceding the epidemic (1399) illustrates the determinants for excess epidemic mortality. Among all of them, 16.2% were dead in the year of epidemic. Among those first registered in the year before the plague, 28.7% were dead. This figure rises to 33% if they were low taxpayers, to 34.3% if they had no ties with other persons within Dijon and to 39.6% if they had a place name. This profile is very suggestive of a poor recent emigrant to the city.

### The 1438–1439 epidemic: Mortality of heads of household and deaths of poor individuals

During the first year of the epidemic, the death rate was lower in the case of the more recently registered (3.6% and 4.7% for those first registered 1 to 3 years prior and 4 to 9 years prior, respectively, compared to 8.4% and 7.7% for those first registered 10 to 32 years prior and 33 to 60 years prior, respectively; N = 224, N = 451, N = 825 and N = 117, respectively; chi-square test; p = 0.0036). During the second year of the epidemic, the death rate was not significantly influenced by time since registration (13.2% for those recently registered compared to 10.9% for those more long-term registered; N = 288 and N = 1,381, respectively). In neither of the two years of the epidemic did the low taxpayers die at a higher rate than their fellow citizens (6.4% compared to 5.5% in 1438 and 11.8% compared to 10.5% in 1439; N = 978, N = 805, N = 920 and N = 749, respectively).

Thus, the heads of household registered during the three years preceding the 1438–1439 epidemic appeared to have been more resistant than those registered during the three years preceding the 1400 epidemic. However they did not differ according to the criteria that could be tested from our database. Their total numbers were comparable (**S5 Table**). Both groups included a higher proportion of low taxpayers than the more long-term registered heads of household of the period (1.86 and 1.74 times, respectively). Between both groups, the proportion of those without demonstrable ties within Dijon was in the same range (79.7% and 77.2%, respectively).

However in 1438 an exceptional mortality was reported among poor individuals, nonregistered as heads of household **(S16 Text)**. Their deaths, not recorded in tax registers, probably reflected the impact of the epidemic as they occurred in the vicinity of the cluster of heads of household with higher mortality **(S3 Fig)**.

### After the epidemics: Demographic recovery and poor newcomers

In the years immediately following the 1438–1439 epidemic, the 1440s were characterized by a marked increase in the number of households: three years after the epidemic, the number of households had increased by 32%. In contrast, the early 1400s were characterized by a lack of recovery following the 1400 epidemic: three years after the epidemic, the number of households had decreased by 1% **(S17 Text)**.

**Fig 7:**
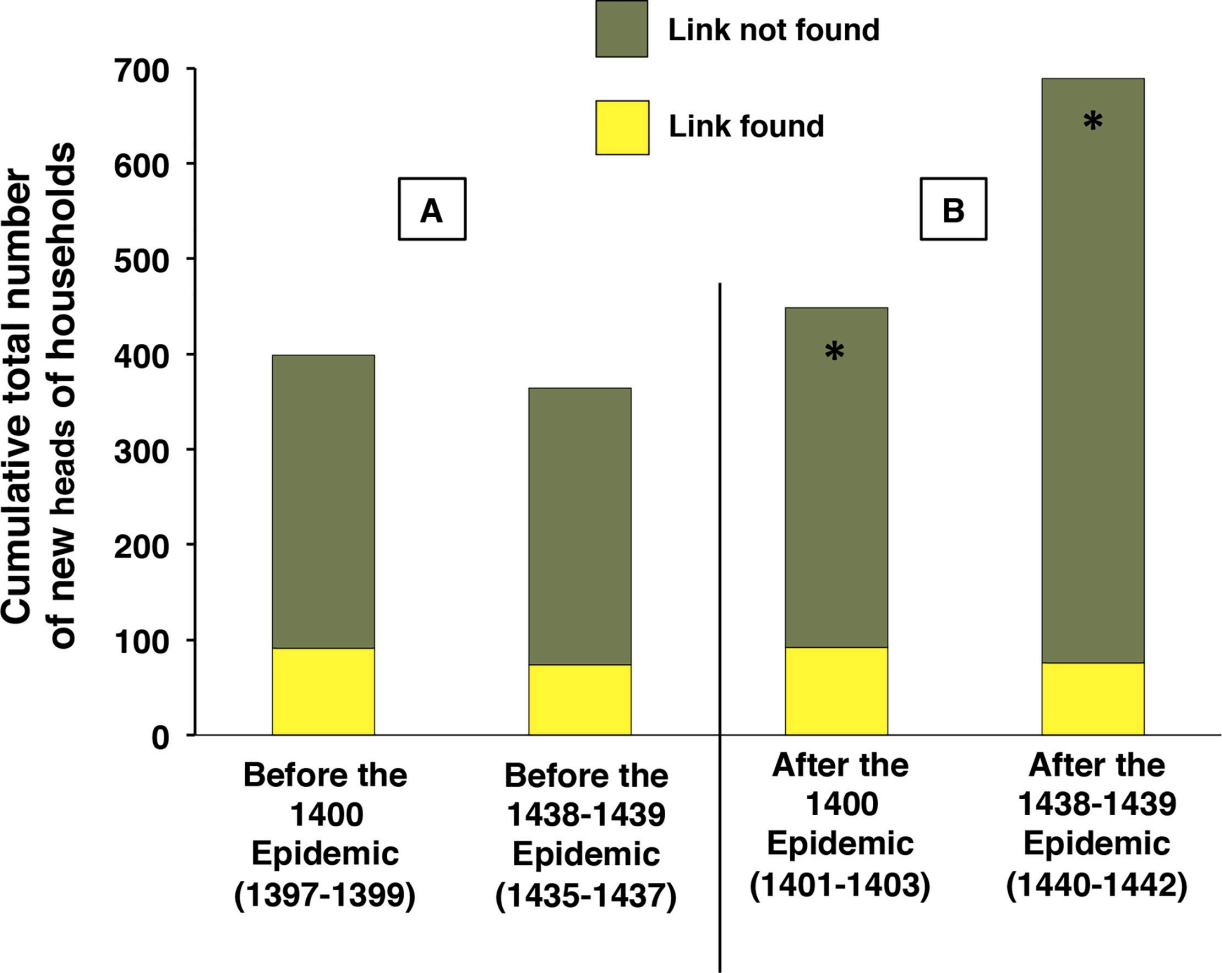
New heads of household according to their links with individuals within Dijon before and after the 1400 and 1438–1439 epidemics. Panel A: Before the epidemics; Panel B: after the epidemics. Link: A link with a person in Dijon at the time of first registration of the new head of household. The numbers in each group are indicated in **S5 Table**. * chi-square test; p < 0.0001.

This difference in the demographic evolution is paralleled by differences in new heads of household, registered during each of the three years following epidemics (Fig 7B). After the 1438–1439 epidemic, new heads of household were more numerous than after the 1400 epidemic. This difference was the result of a clear-cut increase in the number of heads without demonstrable ties within Dijon. In contrast the number of those with a demonstrable link with individuals within Dijon did not vary between the two post-epidemic periods (Fig 7B) and was comparable to the figures observed before the epidemics (Fig 7A).

In addition, the new heads of household registered after the 1438–1439 epidemic included a higher proportion of low taxpayers than did their counterparts registered after the 1400 epidemic (89% and 76%, respectively; chi-square test; p < 0.0001; N indicated in **S5 Table**). Their professions could not be compared because professions were underreported in the early fifteenth century registers.

These results strongly suggest that poor, recent emigrants to the city played a critical role in the demographic recovery that followed the 1438–1439 epidemic.

## Discussion

Here, we show the singularity of probable emigrants to a medieval city, their contribution to the death toll of an early post-Black Death plague recurrence, as well as their contribution to the post-epidemic demographic recovery.

We based our study on the *marcs* registers that annually listed the solvable heads of household in Dijon while recording those who were newly registered and those who died or left during the preceding year. To exploit the continuity of the *marcs* registers over time, we added 50 annual registers to a single database, managed by a specific program based on the establishment of connections between annual entries of households in the tax registers [28]. This provided the following information about the heads of household who faced the 1400 and 1438–1439 epidemics. (i) Their mortality. (ii) Their tax level, indicative of their wealth status, as well as the reason for the exemption of the privileged and very poor heads of household who were not subject to the tax and enlisted. The approximately 50% of heads of household taxed at the lower level, although they do not fully account for the poorest inhabitants, are probably representative of the underprivileged population. (iii) The time elapsed between the epidemic and the heads' first registration, concretizing the fact that they belonged to the urban community. The registration of newly settled heads of household reveals their time of settlement, although the two were probably not strictly synchronous, depending on fiscal practices and on the head's wealth status.

Limitations were imposed on this study by the characteristics of our source documents.

The documents do not provide information on the age nor on the cause of death. However the large numbers of deceased during the three selected years of study indicate that most of the deaths were victims of a communicable disease during the years of these epidemics [5, 6]. In contrast to a number of other source documents [14, 20, 22, 33], the demographic information at our disposal did not include other members of the family. We also lacked information about those poorer inhabitants who were not registered as heads of household. We could not analyze the influence of gender on plague-related mortality because female heads of household were not representative of the population of women. However, our goal was not to describe the absolute demographic effect of the epidemic, but to delineate the impact of a recent settlement on mortality. As far as we know, this factor has not been taken into account in previous studies.

The series of registers, despite their exceptional continuity over time, have three interruptions, generating limitations for statistical analysis. The analysis was performed by two different methods, with similar results.

Our methodology was based on highlighting links between different source documents to identify individuals, as already realized in the case of the Nonantola plague [22]. The large number of household entries required the design of an original program the limitations of which should be discussed. In view of the variability of medieval family names and spellings, the links established by the program between entries within distinct annual registers in order to define a unique individual may be subject to errors. The global likelihood of the chronology proposed for individual heads of household is supported by the followings. (i) During years without an epidemic, the rate of death was lower for the probably younger recently registered heads and higher for the most long-term registered heads. (ii) Among these latters, the size of groups formed according to the time since registration decreased regularly over time (**S2 Fig**).

During the 1400 epidemic, being recently registered represented a determinant for higher mortality that was clear-cut for heads of household first registered in the three years preceding the epidemic and at its maximum for those first registered in 1399. This is demonstrated by two parameters. (i) The one-year fate of the heads of household present in the year preceding the epidemic and (ii) a crude death rate based on the proportion of deaths in relation to the number of entries in the register for the "year of the plague". In contrast to their higher death rates during the epidemic, recently registered heads died at lower rates than the more long-term registered in other ("regular") years. The rate of absence of recently registered heads of household was also higher than that of more long-term registered heads. However, this pattern was also observed during "regular" years, reflecting the previously documented and permanently precarious situation of newly registered heads [34], a sizeable proportion of whom probably returned to their villages of origin after a few years as workers in Dijon. Thus, in 1400, the recently registered heads of household displayed a selective vulnerability to the epidemic.

Our registers provide no information about age that was reported to influence mortality during historic plague epidemics [22, 35–37]. We thus searched for additional determinants of excess epidemic mortality among the vulnerable population of recently registered heads of household. Neither the location of their homes nor their professional activities, which we previously showed to play a role [29], seemed to be relevant. Most of them had a low wealth status as reflected by their lower taxation rate for the *marcs* tax.

The influence of poverty or low socioeconomic status on plague-related mortality is apparent in most studies of preindustrial plagues [3, 14, 31, 38, 39], although the influence varies in the long-term historical perspective [3, 40]. In confirmation of our previous results [29], here we show that in 1400, taxpayers with low *marcs* tax rates displayed a higher mortality than their fellow citizens. However, multivariate analysis indicated that the effect of a low wealth status on epidemic mortality was conditioned by the time elapsed between the first registration and the epidemic. Low taxpayers died in greater numbers only when they were recently registered, and did so at almost twice the rate when they had first been registered one year prior to the epidemic and similarly when they were not recently registered heads of household. In addition, time since registration had no impact on the death rate of non-low taxpayers. Thus, the highly vulnerable individuals were recently registered low taxpayers, whereas neither a low wealth status nor a recent registration was a detectable factor of fragility when isolated.

A new head of household could be a member of a family already settled in Dijon or a newcomer. A population enriched by newcomers was selected on the basis of the lack of familial ties with other persons within the city. Mortality was higher in the subgroup of recently registered individuals without familial links. As a number of links were related to legacies, family links were less often detected for the less affluent, which led us to perform a separate analysis for low taxpayers. When the analysis was restricted to low taxpayers among recently registered heads of household, the death rate was still higher among those without links. This suggests that, at a comparable wealth status, potential newcomers were more vulnerable to the epidemic. However our approach is subject to a number of uncertainties. The links highlighted by the program used to select heads of household with and without ties with other persons within Dijon were obviously not exhaustive and were submitted to bias, such as the already mentioned affluence. We cannot exclude that persons already present in Dijon and newcomers differed by other factors that reduced epidemic vulnerability to the benefit of the former, such as age and/or previous exposure to the disease that may vary between urban and countryside environments [41]. Other confounding factors that cannot be tested from our data may have influenced the mortality analysis, such as a different consideration by the tax authorities for the entry in the register of those with and without links and/or a variation in tax practice during or after epidemics.

Nevertheless, our data are in line with recent results showing that migrants may be excessively susceptible to epidemic mortality. This is the case of plague victims whose lack of registration by an inheritance bureau suggested that they were rural refugees fleeing the Black Death in Cairo [21] and of most victims of fifteenth- to sixteenth-century epidemics in Milan whose place names suggested a recent origin from surrounding villages [19]. Among remains of Black Death victims in London submitted to isotopic analyses, 5 out of 30 probably originated from distant portions of Britain [23]. We extend these data by showing that the more vulnerable population was affected by multiple frailty determinants that probably interacted to weaken the victims of the epidemic. Thus, a poor wealth status was detrimental only for recent emigrants, which could help to reconcile contradictory results about the influence of poverty on mortality during historical plagues. To our knowledge, the interaction of frailty factors, although widely accepted in the case of present-day infection outbreaks [25], has not been quantified in historical epidemics. In addition, the weight of population movement should be considered among the cultural aspects that may influence plague mortality in historical contexts [36].

The pattern was different during the two-year mortality crisis of 1438–1439 [6, 29, 30], which could correspond to a recurring epidemic or reflect a succession of two different diseases. In 1438, an epidemic suggestive of influenza raged through Europe [42], though in Dijon the spatial characteristics of mortality were compatible with a water-borne disease [29]. Neither in 1438 nor in 1439 did recently registered heads of households die in excess. Although heads of household who were recently registered in the years of 1438–1439 and in the year 1400 exhibited a different relative susceptibility during the epidemic, they shared several features: their numbers were comparable and they included comparable proportions of low taxpayers and of persons without ties within Dijon.

This different susceptibility of newcomers may be attributable to the previously mentioned differences in the epidemics [29, 42] or to the additional impact of the famine of the 1430s [31] as a confounding factor. Alternatively, this difference may reflect an acquired resistance of the newcomers, similar to that evidenced in the post-Black Death population of London [43]. In the mid-fifteenth century countryside around Dijon, this could have been a consequence of the major famine [31] in the years preceding the 1438–1439 epidemic. Those individuals able to successfully settle in the capital city of the dukedom might have been among the most resistant of potential emigrants. A non-mutually exclusive hypothesis is that a sizeable portion of poor emigrants moved to the city in search of means of subsistence, as already shown about rural British individuals in response to the crises of the fourteenth century [44]. In Dijon, this fragile population was housed in a hospital, in the immediate vicinity of the area of high mortality of heads of household [29]. These unfortunate residents did not settle as heads of household and were removed from our analysis, potentially explaining the differences between the fates of recently registered individuals facing the 1400 epidemic and those facing the 1438–1439 epidemic.

These two epidemics were followed by contrasting dynamics of recovery, providing an opportunity for an analysis of the role of potential newcomers in the post-epidemic period. Population movement, although poorly studied after historical epidemics, was reported as the preponderant factor of repopulation after a devastating epidemic of the late sixteenth century [45]. In Dijon, after the first epidemic, the number of households declined whereas after the second epidemic, in a context of diminishing armed conflicts and of returning prosperity, the number increased. As expected, new heads of household were more numerous in the years following the 1438–1439 epidemic, a difference entirely explained by the higher number of low taxpayers and of individuals who did not have demonstrable links within Dijon. This strongly suggests that poor newcomers played a key role in the post-epidemic population growth that took part in the urban expansion of the late fifteenth century.

## Conclusion

This work is based on an original program that establishes and exploits links between historical source documents. This allowed the establishment of a database including heads of household in Dijon over a period of seventy-two years. During the deadly plague epidemics that affected Europe half a century after Black Death, we identified among the heads of household a highly vulnerable subgroup, corresponding in all probability to recent emigrants to the city. In this group, recent settlement was combined with two factors of fragility, a low wealth status and an absence of a demonstrable familial links within the city, which were not sufficient for influencing mortality when they were isolated. However, in mid-fifteenth century Dijon, the higher susceptibility of recent emigrants, possibly those who were selected by their survival of prior mass starvation, was not apparent. These emigrants might have reinforced the share of the city's exogenous population. In the following years, in a more favorable economic and political context, poor newcomers played a critical role in the resilience of the city after epidemics.

## Supporting information

S1 Text Historical sources. (PDF)

S2 Text Database and heads of household as persons. (PDF)

S3 Text Heads of household suitable for mortality analysis. (PDF)

S4 Text Persons who were not heads of household. (PDF)

S5 Text Registered heads of household and actual population. (PDF)

S6 Text Absent but still alive. (PDF)

S7 Text One-year follow-up. (PDF)

S8 Text Guidelines for the analysis of time since registration. (PDF)

S9 Text Financial burden of the *marcs* tax. (PDF)

S10 Text Exempted from the *marcs* tax. (PDF)

S11 Text Persons and relationships. (PDF)

S12 Text Data extraction. (PDF)

S13 Text Mortality of those registered the longest. (PDF)

S14 Text Home location of the recently registered heads of household in 1400. (PDF)

S15 Text Professions of the recently registered heads of household in 1400. (PDF)

S16 Text The deaths of poor individuals in 1438. (PDF)

S17 Text Post-epidemic demography. (PDF)

S1 Table Folios 1r and 1v of the year 1400 register (export from the database). (PDF)

S2 Table Actual numbers in Fig 2. (PDF)

S3 Table Actual numbers in Fig 3. (PDF)

S4 Table Parameters of the Binomial GLM dead/nondead ~ wealth * time since registration. (PDF)

S5 Table Actual numbers in Fig 7. (PDF)

S1 Fig Folio 1r of the year 1400 register (source document). (PDF)

S2 Fig Death rate of the most long-term registered heads of household. (PDF)

S3 Fig Cartography of mortality in 1438. (PDF)
