# Supplementary material for: Mortality and demographic recovery in early post-black death epidemics: Role of recent emigrants in medieval Dijon
Source: PLoS One. 2020 Jan 22;15(1):e0226420. doi: 10.1371/journal.pone.0226420 (PMC6975534; doi:10.1371/journal.pone.0226420)
Supplement: S1 Text — (PDF) [file pone.0226420.s001.pdf]

S1 Text. Historical sources

The *marcs* tax was established at the end of the 12th century as an annual global compensation for the granting of a city chart by the duke. From 1284 the global tax was replaced by an individualized contribution paid by the solvent heads of household members of the urban community. For each head of household its level was established and annually revised according to his/her assets within the city, resulting in annual registers (*marcs* registers) enlisting those submitted to the tax as well as a number of exempted from it. Among these registers 69 are preserved, dated from 1357 to 1501 [*Archives Départementales de la Côte d'Or* (ADCO), B11483-B11501].

Each annual register appears as the updating of the previous one. Those present on the previous year were transcribed. Those no more present were also transcribed with the indication of their status (dead or absent and in this case the reason for the absence could be indicated). Newly registered heads of household were added. Additional information likely to alter the tax level could be included and provide information about person(s) related to the head of household such as marriage, death of the spouse, distribution of the tax between the heirs of a deceased head of household. The updating was performed between February and March. At this time, most of the deaths, absences and new registrations had occurred during the previous calendar year. Thus, changes to a given register approximately correspond to the cumulative events of the previous year. For sake of clarity, the registers were identified in this work by reference to the year they covered rather than to the year of updating. As an example, the register updated in 1401 was referred as the "year 1400 register" because it reflects the death toll of the 1400 epidemic.

Other sources, used to enrich the information on heads and members of households were registers for non-annual taxes in the same period of time [ADCO, B11571, B11574, B11577, B11584; *Archives Municipales de Dijon* (AMD), L121-L123, L152], notary records [ADCO,

B11259, B11270, B11274, B11276, B11278, B11290, B11307, B11315, B11319, B11364, B11373, B11380], tax relief requests [AMD, L638-L645], inventories of goods [ADCO, B II 356/1-5] and conviction records [ADCO, B II 362/01].
