# Supplementary material for: Mortality and demographic recovery in early post-black death epidemics: Role of recent emigrants in medieval Dijon
Source: PLoS One. 2020 Jan 22;15(1):e0226420. doi: 10.1371/journal.pone.0226420 (PMC6975534; doi:10.1371/journal.pone.0226420)
Supplement: S2 Text — (PDF) [file pone.0226420.s002.pdf]

S2 Text. Database and heads of household as persons

The database was previously created by two of us from this unique historical source and it was enriched with information from other documents. The original program was based on an adjustment of the database management application *Quatrième Dimension*, specially designed by one of us (HL) for a previous study of the *marcs* registers performed by another of us (AG) [28]. Later, the program was migrated to a dedicated program called "Jehan" developed in C/C++ from the free modules SQLite and wxWidgets. The prerequisite was introducing into the database as much of the information obtained from the annual registers in relation to the households as basic units as possible. The information had to be organized to allow queries for transversal and longitudinal studies.

Performing longitudinal studies required that annual entries of households were converted into corresponding heads of household identified as individuals (or persons) and registered for one or several years. By taking into account characteristics such as name (analyzed in detail in order to take into account its possible variations), tax level, street of residency, relative position in the street (reflected by the order in the enlistment, that is in most cases repetitive from year to year), profession, the program recognized and organized links between the annual entries of households in the registers. On the basis of names and family ties, the program realizes connections within the whole succession of registers. Tentative persons were individualized on the basis of these links. In most cases, the analysis of links allowed to associate annual household entries. In complex or uncertain cases, the investigator controlled the likelihood of persons (if necessary by reexamining the source document) and alterations could be introduced. Such alterations were performed in the course of a previous work [28] and the database was not modified for the present work.

Interactive files are generated by the program: Contributions (providing the information corresponding to the entries in the annual registers); Households (providing the information

about each head of household individualized as a person, such as gender, year of first registration, year of disappearance); Persons (providing the information about each person (whether head of household or not), such as gender, years of first and last quotation, year of death if relevant); Annuities (providing extensive information about a household on a given year, such as presence codification, tax level on the year and on the previous year, persons linked, profession...); Names (allowing to select names according to categories, such as place names); Years; Streets; Professions; Situations (198 types of information about death, reason for absence, reason for exemption, inheritance, disabilities, etc.).

In order to add an annual entry from the register to the database the program adds a record to the Contributions file with the information not connected to a particular person in the household (amount of tax, street, year of the register...). It then draws up the list of this/those person(s) and their relationships without knowledge of their presence in the database. Finally, in order to identify them, the program calculates (on the basis of the names and/or of the relationships) a proximity index with those persons already present in the database. If one of them is close enough it is identified and the information concerning this person is enriched. If this is not the case a new person is added to the database. If required, the relationships between persons present in the database are updated.

An individual can be selected in the database from his/her name and/or the link he/she has with an already identified person. Each individual head of household identified as a person was endowed with: an ID number, a name consisting of his/her first name (that does not vary throughout the registers) and a last name (standardized in order to take into account that, during the course of the years, the last name often varied in spelling and sometimes in denomination), a profession (when indicated), a year of appearance in the registers and a year and mode of disappearance from them. From the 50 annual registers, 108,193 entries of

households were included in the database. Taking these entries into account, the program proposed 13,001 individual heads of household with a defined duration of registration.
