# Supplementary material for: Mortality and demographic recovery in early post-black death epidemics: Role of recent emigrants in medieval Dijon
Source: PLoS One. 2020 Jan 22;15(1):e0226420. doi: 10.1371/journal.pone.0226420 (PMC6975534; doi:10.1371/journal.pone.0226420)
Supplement: S3 Text — (PDF) [file pone.0226420.s003.pdf]

S3 Text. Heads of household suitable for mortality analysis

A number of the 13,001 individual heads of household did not correspond to persons suitable for demographic analysis: 38 households corresponded to houses such as religious institutions; 46 households were collective such as "the heirs of"; 39 households were anonymously identified by a function such as "the vicar of"; 4 households were named "a household" without additional information. They were not taken into account for the present work. The 12,874 individual heads of household eligible for mortality analysis corresponded to a total of 104,500 annual entries of households in the 50 registers.
