# Supplementary material for: Mortality and demographic recovery in early post-black death epidemics: Role of recent emigrants in medieval Dijon
Source: PLoS One. 2020 Jan 22;15(1):e0226420. doi: 10.1371/journal.pone.0226420 (PMC6975534; doi:10.1371/journal.pone.0226420)
Supplement: S4 Text — (PDF) [file pone.0226420.s004.pdf]

**S4 Text. Persons who were not heads of household**

Persons who were not heads of household were mentioned in the *marcs* registers either directly, such as a deceased wife whose property is distributed, the heirs of a deceased head of household, the homeowner or the employer of a head of household, or indirectly when a head of household is qualified as the son of (...) or the widow of (...). Such persons were introduced by the program in the database concomitantly with the head of household with whom they had a link.

A number of persons not mentioned in the *marcs* registers were also introduced in the course of the work that led to the creation of the database (the goal was to define the socio-economic and professional characteristics of the population of Dijon [28]). Only 128 out of the 1,980 non-heads of household persons were introduced in the database without being mentioned in the *marcs* registers. They corresponded to 114 entries among which 47 were quoted in other tax registers together with a head of household also submitted to the *marcs* tax. The other entries corresponded to persons related to heads of household quoted in published works about Dijon notables and traders, in tax relief requests, in inventories after death or in notary records.
