# Supplementary material for: Mortality and demographic recovery in early post-black death epidemics: Role of recent emigrants in medieval Dijon
Source: PLoS One. 2020 Jan 22;15(1):e0226420. doi: 10.1371/journal.pone.0226420 (PMC6975534; doi:10.1371/journal.pone.0226420)
Supplement: S5 Text — (PDF) [file pone.0226420.s005.pdf]

S5 Text. Registered heads of household and actual population

A comparison with contemporary registers for occasional taxes levied on every head of household in the city indicates that the heads of household enlisted in the *marcs* registers are representative of the taxpayers in Dijon. For year 1375, the *marc* register enlists 2,438 heads of household present, compared to 2,469 in another tax register of the same year [28, p374]. For year 1446, the corresponding figures are 2,071 in the *marcs* register and 2,116 in the register of the tax levied for the fortress [28, p379].

The relationship between the number of households and the number of inhabitants varies according to places and demographic conditions [Blockmans W, Dubois H. [Times of crises (14th and 15th centuries)], in Bardet JP and Dupâquier J, editors [History of populations in Europe: 1 From the origins to the beginnings of demographic revolution]. Paris: Fayard; 1997, p 208. French]. A 3 to 5 factor is often proposed to evaluate the latter from the former. The estimate of a 10,000 population in the 14th to 15th century Dijon (obtained by confronting multiple documents) is based on such an assumption [Humbert F. [Municipal funds in Dijon from the mid-14th century to 1477]. Paris: Les Belles Lettres; 1961, p 24. French].

The proportion of very poor heads of household, vagabonds and beggars left aside from fiscal documents has been estimated to a few percent in medieval Paris [Geremek B. The margins of society in late medieval Paris. Cambridge: Cambridge University Press; 1987, translated from Polish]. In Dijon, a register of households performed in year 1431 divided the population into classes according to socioeconomic status. Among the 1,800 heads of household enlisted, 472 were qualified as beggars ("begging one's bread") and belonged to the lower class: 38% of them were recorded in the nearest *marcs* register in time (year 1428), compared to 68% of those belonging to the immediately higher class ("miserable") [28, pp 375-376]. This suggests that approximately 30% of the 472 beggar heads of household were not taken into account in the contemporaneous *marcs* registers (corresponding to 5 to 10% of our dataset population).
