# Supplementary material for: Mortality and demographic recovery in early post-black death epidemics: Role of recent emigrants in medieval Dijon
Source: PLoS One. 2020 Jan 22;15(1):e0226420. doi: 10.1371/journal.pone.0226420 (PMC6975534; doi:10.1371/journal.pone.0226420)
Supplement: S6 Text — (PDF) [file pone.0226420.s006.pdf]

S6 Text. Absent but still alive

Heads of household could be transiently absent and present in a subsequent register, in most cases in the next 1 to 3 years (they accounted for 21% of absent during the three years of epidemics). Alternatively, their living status could be identified from an indication of the reason for their absence: they could be still present in Dijon but no more heads of household, because of an entry to the service of a master, into a hospital, into a monastery or of a new wedding in the case of a widow head of household (5% of absent during the three years of epidemics); they could have left the city, with indication of the new place of residency (6% of absent during the three years of epidemics).
