# Supplementary material for: Mortality and demographic recovery in early post-black death epidemics: Role of recent emigrants in medieval Dijon
Source: PLoS One. 2020 Jan 22;15(1):e0226420. doi: 10.1371/journal.pone.0226420 (PMC6975534; doi:10.1371/journal.pone.0226420)
Supplement: S7 Text — (PDF) [file pone.0226420.s007.pdf]

S7 Text. One-year follow-up

For one-year follow-up, two lists of heads of household were established. (1) Those present on the year preceding the epidemic (year 0). (2) Those enlisted on the year of epidemic (whether present, dead or absent) and registered at least one year earlier (year 1). From the year 0 list, the heads of household not found on year 1 were taken into account as lost to follow-up. From the year 1 list, a small number of heads of household were excluded because they were not enlisted on year 0 (they had been registered earlier but were transiently absent on year 0). The heads of household appearing on both year 0 and year 1 lists were selected for analysis and their fate on year 1 was taken into account.
