# Supplementary material for: Mortality and demographic recovery in early post-black death epidemics: Role of recent emigrants in medieval Dijon
Source: PLoS One. 2020 Jan 22;15(1):e0226420. doi: 10.1371/journal.pone.0226420 (PMC6975534; doi:10.1371/journal.pone.0226420)
Supplement: S8 Text — (PDF) [file pone.0226420.s008.pdf]

S8 Text. Guidelines for the analysis of time since registration

The first registration could not be precisely determined for heads of household whose first registration took place either in the first register (1375) or in a register immediately following a period where registers were missing (1393, 1418 and 1433). For single variable analysis, the values of time since registration were grouped into categories as shown in *Fig 1B* with the following guidelines. (i) Individualize the shorter times of years 1, 2 and 3, for separate or grouped analysis; (ii) Take into account the impossibility of splitting the time periods starting in 1375, 1393, 1418 or 1433; and (iii) Delineate categories of roughly equal numbers of heads of household. For multivariate analysis, time between registration and the 1400 epidemic was treated as a continuous variable (categories starting in 1375, 1393, 1418 and 1433 were attributed a rank corresponding to the median of the category).

For the analysis of the impact of time since registration on mortality, those first registered in the year of interest (the "0 year" time since registration) were excluded. Indeed, with few exceptions, when the clerk registered new heads of household, his goal was to complete the list of taxable households and he was prone to consider only living persons. During the three years of epidemic, among heads of household with a "0 year" time since registration only 1 death (out of a total of 620) was reported, in 1400. In all probability, the clerk did not register the potential new heads of household who had succumbed to the epidemic during their first months of presence. When mortality was considered, these heads of household first registered on the year under study were not comparable to heads of household registered previously. The influence of time since registration on mortality was thus analyzed for those registered before the year of epidemic, being registered since at least one year.

In the same line, absent were exceptionally taken into account for new registration: during the same three years mentioned above, only 2 absent (among 267) were registered as new heads of household.
