# Supplementary material for: Mortality and demographic recovery in early post-black death epidemics: Role of recent emigrants in medieval Dijon
Source: PLoS One. 2020 Jan 22;15(1):e0226420. doi: 10.1371/journal.pone.0226420 (PMC6975534; doi:10.1371/journal.pone.0226420)
Supplement: S9 Text — (PDF) [file pone.0226420.s009.pdf]

S9 Text. Financial burden of the *marcs* tax

The total amount of the *marcs* tax was marginal in the duke's annual income [Rauzier J. [Budget and management of a principality in the 14th century: the Burgundy dukedom of Philip the Bald]. Comité pour l'histoire économique et financière de la France, editor. Paris: Imprimerie Nationale; 1996, p 245 & 699. French]. The information provided on the heads of household by the registers was probably of higher value for the power than the tax itself. This probably explains why non-members of the commune were included in the registers (although they were not liable for the *marcs* tax) such as Jews (before they were expelled from the French kingdom in 1394), nobles and a number of clergy members.

Although the very poor were not (or no more) recorded in the registers, most low taxpayers charged at the 1sol lower limit of the *marcs* tax can be considered as members of the poor population of Dijon. Their proportion corresponds to that of the taxpayers who were considered below poverty line in Lyon in the same period [Gonthier N. [Lyon and its poor in the middle ages (1350-1500)]. Lyon: L'Hermès; 1987, p 40. French] and of the day-to-day workers in the medieval town [Desportes P. [The city] in Favier J, editor [Medieval France] Paris: Fayard; 1983, p 201-215. French].

A 1sol annual tax can be put in perspective with the daily wage of a winegrower (winegrower is the more frequently mentioned profession for low taxpayers). This salary amounted 1 to 2 sols at the end of the 14th century [Stella A. [The social profile of winegrowers in northern Burgundy from the 14th to the 18th century]. *Bulletin du Centre Pierre Léon d'histoire économique et sociale*. 1996;3-4: 79. French] and 2 to 3 sols in the mid-15th century [Tournier C. [Notes about vineyard growing and winegrowers in Dijon between 1430 and 1560]. *Annales de Bourgogne*. 1952;24: 151-152. French].
