# Supplementary material for: Mortality and demographic recovery in early post-black death epidemics: Role of recent emigrants in medieval Dijon
Source: PLoS One. 2020 Jan 22;15(1):e0226420. doi: 10.1371/journal.pone.0226420 (PMC6975534; doi:10.1371/journal.pone.0226420)
Supplement: S10 Text — (PDF) [file pone.0226420.s010.pdf]

S10 Text. Exempted from the *marcs* tax

A number of registered heads of household were exempted from the *marcs* tax, either as members of a group not submitted to the *marcs* tax (nobles, ecclesiastics) or because of an individual exemption, in most cases because of a privilege (officers and providers of the duke, clerks...) and in a minority of cases because of poverty. During the three years of epidemics, nobles accounted for 0.6%, ecclesiastics identified as individuals for 1.1%, exempted by privilege for 3.3% and exempted for poverty for 0.8% of the heads of household present.

Qualified as "beggars" by the clerk, these latters were not representative of a taxation category that would be exempted on a stable basis. Rather, they represented selected heads of household whose insolvability was noticed by the clerk. With few exceptions, the clerk did not make the effort to register them for several years and their ultimate fate was unknown [28, pp 383-385]. Later on, most of them were indicated as absent with a homeless status (*ne tient ni feu ni lieu*). Others were lost to follow-up, and they accounted for most of these latters. Indeed, among the heads of household present in 1399 and lost to follow-up in 1400, all but one had been exempted for poverty: their disappearance from the registers reflected their insolvability rather than an involuntary omission by the clerk.
