# Supplementary material for: Mortality and demographic recovery in early post-black death epidemics: Role of recent emigrants in medieval Dijon
Source: PLoS One. 2020 Jan 22;15(1):e0226420. doi: 10.1371/journal.pone.0226420 (PMC6975534; doi:10.1371/journal.pone.0226420)
Supplement: S11 Text — (PDF) [file pone.0226420.s011.pdf]

**S11 Text. Persons and relationships**

The program highlights the relationships between the 14,981 persons included in the database. Among the 13,001 individual heads of household, 4,193 had a detectable relationship with one or several person(s) in the database, among other heads of household or among the 1,980 non-heads of household. The program provided the ID numbers of the persons concerned, the type of links and the hierarchy for each link. In case of complex or multiple links, or if the date of establishment of the link had to be taken into account, a search was performed in the database, in order to document more precisely the relationship.

The selection of recently registered heads of household with a detectable familial tie with a person in Dijon was based on the following links: parent/child (a relationship often but not always apparent from the name), spouse (that led to son in law when combined with parent), brother/sister, heir/legatee. When the link indicated a marriage between a head of household and another person (usually the wife), it was taken into account only when the related person was already present in Dijon (usually as a the widow of another head of household) and when the marriage took place soon after registration (on the year of registration or the year after).

The links between employee and boss and between tenant/lodger and landlord were not taken into account because we restricted our analysis to familial ties, more indicative of a strong social bond.
