# Supplementary material for: Mortality and demographic recovery in early post-black death epidemics: Role of recent emigrants in medieval Dijon
Source: PLoS One. 2020 Jan 22;15(1):e0226420. doi: 10.1371/journal.pone.0226420 (PMC6975534; doi:10.1371/journal.pone.0226420)
Supplement: S12 Text — (PDF) [file pone.0226420.s012.pdf]

S12 Text. Data extraction

For every year when an individual head of household is registered, the database indicates information specific for the year, such as the native last name, the reference to the page of the register (and its position within it), the street and parish of home, the amount of tax or in case of tax exemption its grounds (reflecting the socio-economic status), the presence, death or absence, whether an absence is temporary or definitive, and (when known) the reason for an absence, etc.

Files of selected heads of household can be generated by queries based on ID number, name, year(s) of registration, tax level, profession, year of first registration, mode of disappearance, etc. Persons linked to a head of household (whether or not they are themselves a head of household) can be selected.

Data counts and analysis can be realized by the program itself or after export in an application.
