# Supplementary material for: Mortality and demographic recovery in early post-black death epidemics: Role of recent emigrants in medieval Dijon
Source: PLoS One. 2020 Jan 22;15(1):e0226420. doi: 10.1371/journal.pone.0226420 (PMC6975534; doi:10.1371/journal.pone.0226420)
Supplement: S13 Text — (PDF) [file pone.0226420.s013.pdf]

S13 Text. Mortality of those registered the longest

During "regular" years, the mortality of recently registered heads of household was lower than that of heads who had been registered longer. We thus tested whether, during these "regular" years, the mortality of the most long-term registered heads of household was higher. To this aim, we compared two groups of long-term registrants (*Fig 1B*). (i) The most long-term registrants, who were present in the first register of our database (1375): their first registration took place at least 25 years before the epidemic. (ii) Less long-term registrants, appearing in the second register up to those appearing in the last register of the same continuous series (1376-1385): their first registration took place 15-24 years before the epidemic.

As shown in **S2 Fig**, during the six years preceding the 1400 epidemic, the most long-term registrants constantly exhibited a higher mortality than the group of less long-term registrants. During the "year of the plague", as already shown above, the death rates were comparable in the two groups. During the six following years, mortality was again higher for the most long-term registrants. A Wilcoxon signed-rank test on the whole time series rejects the null hypothesis of a similar death rate for the two groups ($p = 0.008$), although the two groups peak at a similarly high death rate in 1400.
