# Supplementary material for: Mortality and demographic recovery in early post-black death epidemics: Role of recent emigrants in medieval Dijon
Source: PLoS One. 2020 Jan 22;15(1):e0226420. doi: 10.1371/journal.pone.0226420 (PMC6975534; doi:10.1371/journal.pone.0226420)
Supplement: S14 Text — (PDF) [file pone.0226420.s014.pdf]

S14 Text. Home location of the recently registered heads of household in 1400

The proportion of heads of household whose homes were located within the excess mortality areas [29] was comparable among recently registered and among those having been registered longer (5.2% and 6.6%, respectively; N = 286 and N = 1,594, respectively).
