# Supplementary material for: Mortality and demographic recovery in early post-black death epidemics: Role of recent emigrants in medieval Dijon
Source: PLoS One. 2020 Jan 22;15(1):e0226420. doi: 10.1371/journal.pone.0226420 (PMC6975534; doi:10.1371/journal.pone.0226420)
Supplement: S15 Text — (PDF) [file pone.0226420.s015.pdf]

S15 Text. Professions of the recently registered heads of household in 1400

In the early 1400s years a profession was indicated for less than half of the male heads of household and this indication was slightly more frequent for the recently registered heads than for the more long-term registered (44.2% and 37.7%, respectively; N = 274 and N = 1,436, respectively; chi-square test; p = 0.043). Recently registered were more often winegrowers (20.7%, compared to 13.1%; N = 121 and N = 541, respectively; chi-square test; p = 0.033) a profession that we previously showed to be associated with a lower epidemic mortality [29]. Otherwise no particularity was apparent in the repartition of their professions except for the absence of prominent officer of the duke or of the city among recently registered heads.
