# Supplementary material for: Mortality and demographic recovery in early post-black death epidemics: Role of recent emigrants in medieval Dijon
Source: PLoS One. 2020 Jan 22;15(1):e0226420. doi: 10.1371/journal.pone.0226420 (PMC6975534; doi:10.1371/journal.pone.0226420)
Supplement: S16 Text — (PDF) [file pone.0226420.s016.pdf]

S16 Text. The deaths of poor individuals in 1438

In 1438 an exceptional number of poor were housed at the Saint Esprit hospital in Dijon and the majority of them died. This is reported as follows by François Calmelet (the last commander of this hospital).

"[The commander] housed in his hospital up to 15 thousand poor during the 1438 year that was desolated by one of the biggest mortalities that had ever felt the people of Dijon. There died ten thousand of these poor..."

Although probably exaggerated, this number of deaths (in the range of the total estimated population of Dijon) suggests that a large proportion of the deceased were refugees from nearby villages fleeing the famine.

[Source: Calmelet F. [History of the masterful monastery and hospital house of the Saint Esprit in Dijon]. Copy kept at Dijon City Library [ms 934], p 56. French. The original document source is quoted as the "new cartulary of this house closed and finished in 1757".]

The Saint Esprit hospital was located along the Ouche River, on the outer side of the eponym gate. The poor who died in the hospital did so in the immediate vicinity of the 1438 cluster of higher mortality among heads of household that we previously evidenced at the confluence of the rivers Ouche, Suzon and Renne [29].
