# Supplementary material for: Mortality and demographic recovery in early post-black death epidemics: Role of recent emigrants in medieval Dijon
Source: PLoS One. 2020 Jan 22;15(1):e0226420. doi: 10.1371/journal.pone.0226420 (PMC6975534; doi:10.1371/journal.pone.0226420)
Supplement: S17 Text — (PDF) [file pone.0226420.s017.pdf]

S17 Text. Post-epidemic demography

The 1400 epidemic inaugurated a prolonged fall in the number of heads of household registered as present in the *marcs* registers, from 1,997 in 1399 to 1,715 in 1400, followed by a continuous decline to a nadir of 1,280 in 1422. The 1338-1439 epidemic resulted in a comparable fall from 1,780 in 1437 to 1,508 in 1439, but was followed by a sustained increase, to 2,071 in the 1446 register, inaugurating the demographic recovery of the second half of the 15th century (3,070 heads of household were present in 1501).
