# Supplementary material for: Mortality and demographic recovery in early post-black death epidemics: Role of recent emigrants in medieval Dijon
Source: PLoS One. 2020 Jan 22;15(1):e0226420. doi: 10.1371/journal.pone.0226420 (PMC6975534; doi:10.1371/journal.pone.0226420)
Supplement: S1 Table — (PDF) [file pone.0226420.s018.pdf]

S1 Table. Folios 1r and 1v of the year 1400 register (export from the database)

Feu ID	Name	Comp	Year	Ctr ID	Street	Parish	Starts	Pres	End	Tax	Tax P	Sit	Professions
1678	Jehan de Binges (2)	MS	1400	01r01	1	5	1375	5	0	3	3		
4815	Huguenin de Saint_Thibault	MS	1400	01r02	1	5	1393	5	0	3	2		corduannier
6075	Jehan Anthoine_	MS	1400	01r03	1	5	1395	0	9000	0	2	mort	fournier
6867	femme Jehan Anthoine_	FS	1400	01r04	1	5	1400	5	0	5	0	no	
4816	Arniote la Lorraine	FS	1400	01v01	1	5	1393	5	0	1	1		
659	Richard le Cousturier de Trichastel	MS	1400	01v02	1	5	1375	5	0	1	1		couturier(), pelletier()
4818	Denisot de Vicherey	MS	1400	01v03	1	5	1393	5	0	1	1		
6710	Sebille la Boiteuse	FS	1400	01v04	1	5	1399	0	2000	0	1	alee	
4820	Viennot Poirot	MS	1400	01v05	1	5	1393	5	0	4	4		
6711	Pierre de Poligny (1)	MS	1400	01v06	1	5	1399	5	0	30	20	pm	
6403	le Bon Jehan	MS	1400	01v07	1	5	1397	0	9000	0	1	MS, mort	
6712	Pierre d’ Ampilly	MS	1400	01v08	1	5	1399	0	2000	0	1	ale	
4822	Guienot de Choye	MS	1400	01v09	1	5	1393	5	0	1	1		
4823	Huguenin de Saint_Leger	MS	1400	01v10	1	5	1393	5	0	1	1		
6868	Jaquin Chevaleret	MS	1400	01v11	1	5	1400	5	0	1	0	no	
6582	Sebille d' Etais	FS	1400	01v13	1	5	1398	5	0	1	1		
4825	Richard Perrot_	MS	1400	01v14	1	5	1393	5	0	1	1		perrier
6232	Guillaume de Saulx (3)	MS	1400	01v15	1	5	1396	5	0	1	0	no, rvnt	
6233	Milot de Montarlot	MS	1400	01v16	1	5	1396	2	2000	0	1	ale	

Legend to the Table:

Feu ID: household ID number. Name: standardized name of the head of household. Comp: composition of the household (FS = female head of household; MS = male head of household). Year: year under study. Ctr ID: contribution identification based on the entry in the register (folio number; side of the page (recto or verso); rank in the side of the page). Street: ID number of the home street in the database. Parish: ID number of the home parish in the database. Starts: year of first registration. Pres: codification for presence (5 = present; 0 = definitively absent; 2 = transiently absent). End: codification for the end on the year of study (0 = no end; 2000 = absent; 9000 = dead). Tax: amount of tax of the year (in sol). Tax P = amount of tax of the previous year (in sol) used to figure out the tax level of non-present. Sit: information on a number of situations (mort = dead; no = new; ale(e) = gone; no, rvnt = comes back). Professions: the indication of profession is poor in the early 1400s registers (known for about 40% of male heads of household; later on it progressively increases (known for more than 80% of male heads of household in the 1440s registers). The recto of the corresponding source document is presented in **S1 Fig** and its verso was previously published in Fig 3 of [29].

Examples:

Jehan de Binges (2) (ID 1678) is a male head of household, is registered as number 1 on the recto of folio 1 of year 1400 register, his home is in street 1 of parish 5, he was present on the first year of the database (first registration on 1375 or earlier), is still present, is taxed at 3 sols (same amount as previous year).

Jehan Anthoine (ID 6075) was a male head of household, registered since 1395 as a baker and taxed at 2 sols. In 1399 he was registered as the third head of household in the first folio. In 1400, he is no more mentioned in the source and is replaced by his widow (see below). He is thus introduced as deceased in the database.

Femme Jehan Anthoine (ID 6867) the widow of Jehan ("la femme feu..." in **S1 Fig**) is first registered in 1400 at the same rank as was Jehan on the previous year (whereas she stands in the third line of the source document, she is attributed CtrID 01r04 in the database to take into account the introduction of Jehan). She will be present as a head of household until the end of the series in 1406.

Sebille la Boiteuse (ID 6710) was a female head of household, is registered as number 4 on the verso of folio 1, was registered since 1399, is definitively absent, left in 1400 and was taxed at 1 sol on the previous year.

Milot de Montarlot (ID 6233) is a male head of household and was registered since 1396. In 1400 he is transiently absent and was taxed at 1 sol on the previous year. The database indicates that he will be back two years later and will be present until the end of the series in 1406.
