# Supplementary material for: Mortality and demographic recovery in early post-black death epidemics: Role of recent emigrants in medieval Dijon
Source: PLoS One. 2020 Jan 22;15(1):e0226420. doi: 10.1371/journal.pone.0226420 (PMC6975534; doi:10.1371/journal.pone.0226420)
Supplement: S2 Table — (PDF) [file pone.0226420.s019.pdf]

S2 Table. Actual numbers in Fig 2

Time since registration	Lost	Absent	Dead	Living	Total
1 year	0	14	39	83	136
2 years	2	11	17	61	91
3 years	1	11	24	73	109
4-6 years	1	12	38	177	228
7-14 years	4	19	97	481	601
15-24 years	3	4	52	317	376
≥ 25 years	6	9	57	384	456
