# Supplementary material for: Mortality and demographic recovery in early post-black death epidemics: Role of recent emigrants in medieval Dijon
Source: PLoS One. 2020 Jan 22;15(1):e0226420. doi: 10.1371/journal.pone.0226420 (PMC6975534; doi:10.1371/journal.pone.0226420)
Supplement: S3 Table — (PDF) [file pone.0226420.s020.pdf]

S3 Table. Actual numbers in Fig 3

Time since registration	Number of dead	Total number of households*
1-3 years	80	336
4-6 years	38	230
7-14 years	97	601
15-24 years	52	376
≥ 25 years	57	453

* The numbers may slightly differ from those in **S2 Table** due to lost to follow-up in 1400 and to transient absences in 1399 (**S7 Text**).
