# Supplementary material for: Mortality and demographic recovery in early post-black death epidemics: Role of recent emigrants in medieval Dijon
Source: PLoS One. 2020 Jan 22;15(1):e0226420. doi: 10.1371/journal.pone.0226420 (PMC6975534; doi:10.1371/journal.pone.0226420)
Supplement: S4 Table — (PDF) [file pone.0226420.s021.pdf]

S4 Table. Parameters of the Binomial Generalized Linear Model dead/non-dead ~ wealth * time since registration

Term	Estimate	CI95% Lower limit	CI95% Upper limit
(Intercept)	0.172	0.116	0.256
Wealth (low taxpayer)	2.011	1.256	3.221
Time since registration	0.993	0.972	1.015
Wealth (low taxpayer): time since registration	0.965	0.935	0.997
