# Supplementary material for: Mortality and demographic recovery in early post-black death epidemics: Role of recent emigrants in medieval Dijon
Source: PLoS One. 2020 Jan 22;15(1):e0226420. doi: 10.1371/journal.pone.0226420 (PMC6975534; doi:10.1371/journal.pone.0226420)
Supplement: S5 Table — (PDF) [file pone.0226420.s022.pdf]

S5 Table. Actual numbers in Fig 7

	Before 1400 epidemic	Before 1438-9 epidemic	After 1400 epidemic	After 1438-9 epidemic
No link found	308	290	357	613
Link Found	91	74	92	76
Total Number	399	364	449	689
