# Supplementary material for: Mortality and demographic recovery in early post-black death epidemics: Role of recent emigrants in medieval Dijon
Source: PLoS One. 2020 Jan 22;15(1):e0226420. doi: 10.1371/journal.pone.0226420 (PMC6975534; doi:10.1371/journal.pone.0226420)
Supplement: S1 Fig — (PDF) [file pone.0226420.s023.pdf]

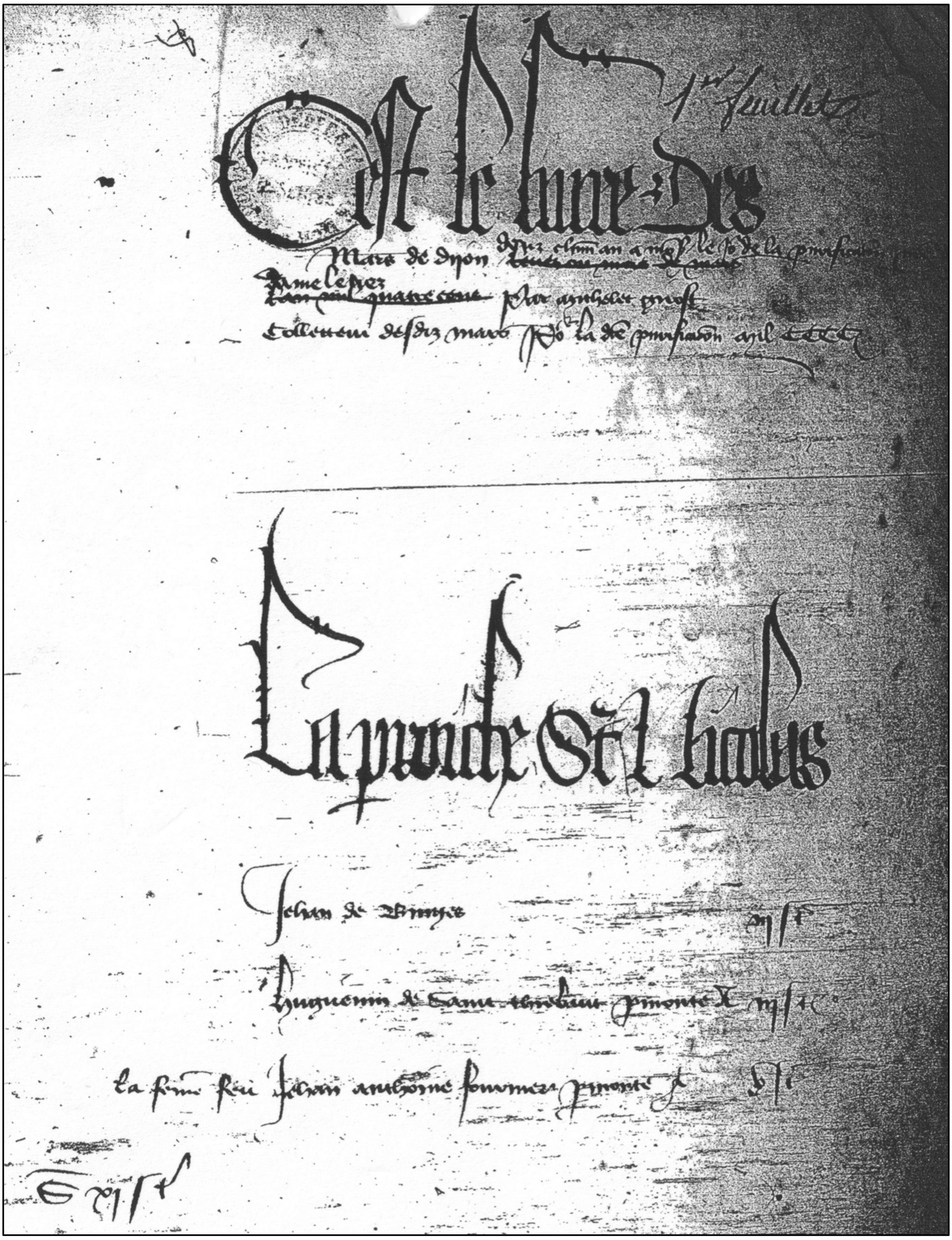

S1 Fig. Folio 1r of the year 1400 register (source document)
After a preamble and indication of the name of the parish
The first 3 enlisted heads of households are mentioned.
The last one corresponds to a deceased and his wife in the database (see **S1 Table**).
Picture reproduced (with improvement in contrast and brightness)
from a personal photograph of a publicly available source document
[ADCO B11490 (as quoted in ***S1 Text****)].*
No previous copyright.
