# Supplementary material for: Mortality and demographic recovery in early post-black death epidemics: Role of recent emigrants in medieval Dijon
Source: PLoS One. 2020 Jan 22;15(1):e0226420. doi: 10.1371/journal.pone.0226420 (PMC6975534; doi:10.1371/journal.pone.0226420)
Supplement: S2 Fig — (PDF) [file pone.0226420.s024.pdf]

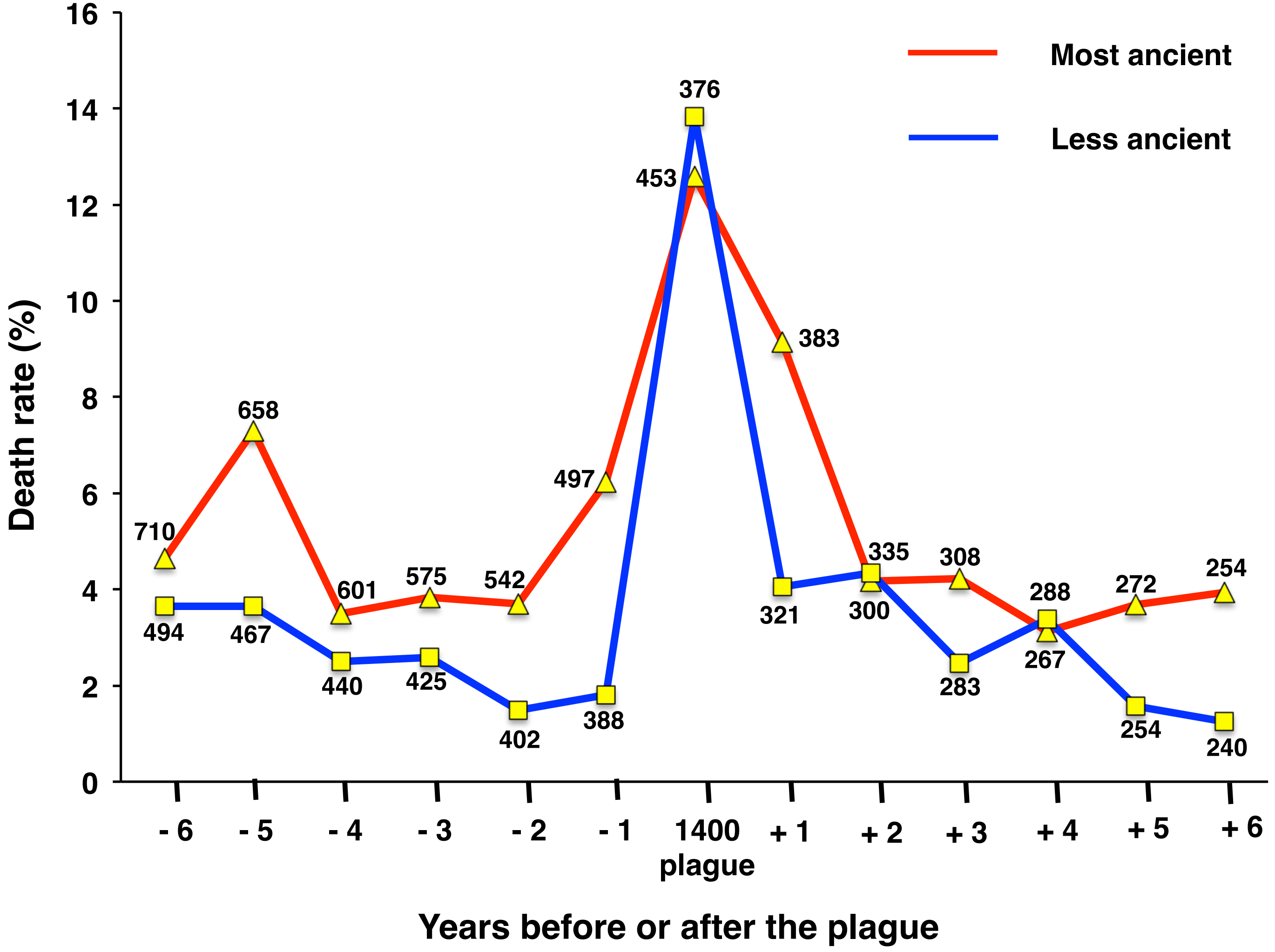

S2 Fig. Death rate of the most long-term registered heads of households

Death rates: number of deaths of heads of household/total number of household entries in the register.

Time since registration ≥ 25 years: red line, triangle marks.

Time since registration of 15-24 years: blue line, square marks.

Annual total for the number of heads of household (which regularly decreases over time) indicated for each group.

*Figure based on personal unpublished data as described in **S13 Text**. No previous copyright.*
