# Supplementary material for: Mortality and demographic recovery in early post-black death epidemics: Role of recent emigrants in medieval Dijon
Source: PLoS One. 2020 Jan 22;15(1):e0226420. doi: 10.1371/journal.pone.0226420 (PMC6975534; doi:10.1371/journal.pone.0226420)

S3 Fig. Cartography of mortality in 1438

Map frame: GIS-based map of Dijon in the 15th century

Yellow area circled in red: over-mortality of heads of households evidenced by spatial analysis [29]

White area circled in red: location of the Saint Esprit hospital

Box: detail of the first preserved map of Dijon (hospital circled in red).

*Figure based on personal data [29 and this work (**S16 Text**)].*

The map of Bredin is a publicly available document kept at the Archives Municipales de Dijon.

No previous copyright.

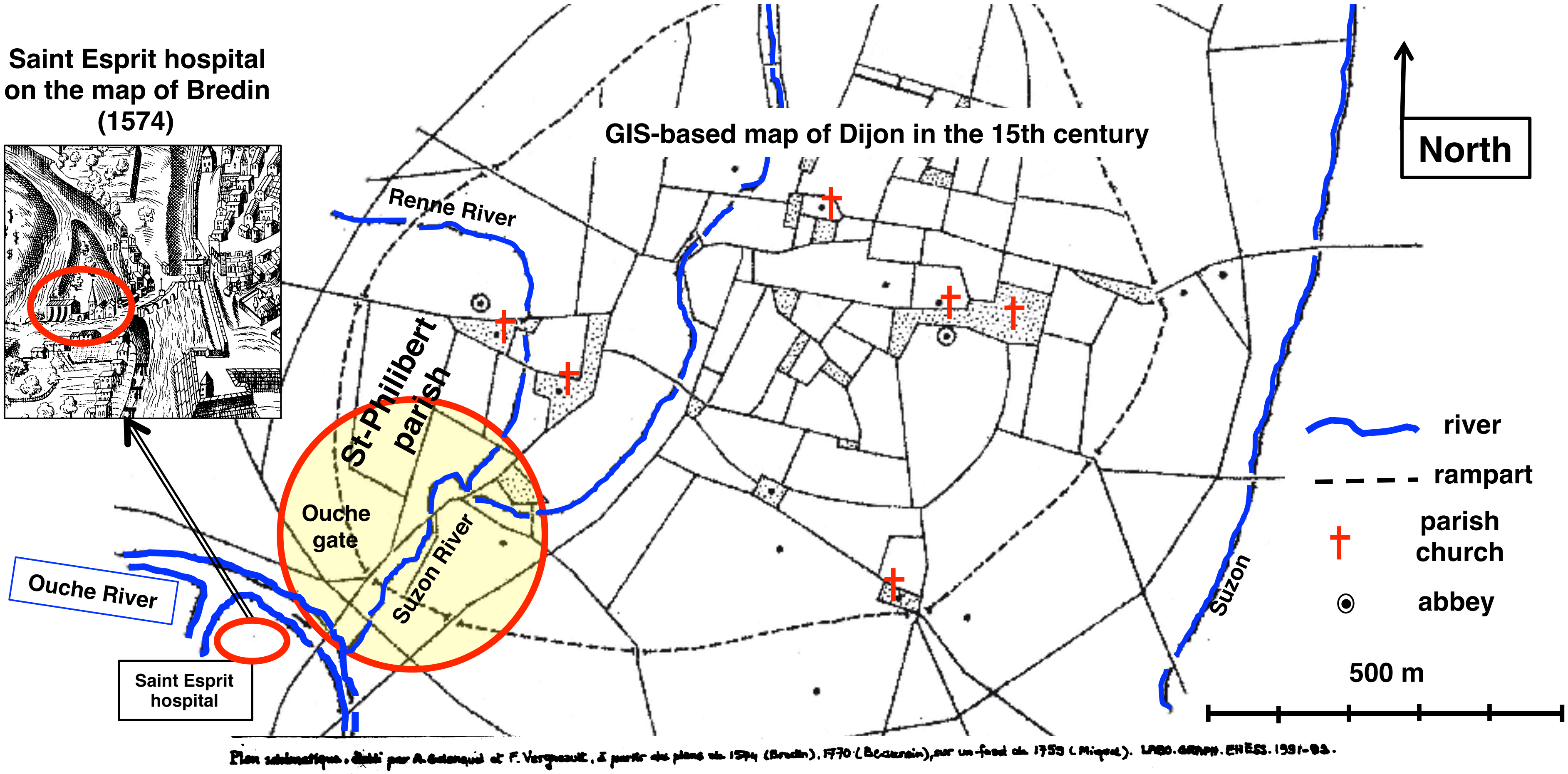

Supplement: S3 Fig — (PDF) [file pone.0226420.s025.pdf]
